# Preliminary Assessment of Criticality Safety Constraints for Swiss Spent Nuclear Fuel Loading in Disposal Canisters

**DOI:** 10.3390/ma12030494

**Published:** 2019-02-05

**Authors:** Alexander Vasiliev, Jose Herrero, Marco Pecchia, Dimitri Rochman, Hakim Ferroukhi, Stefano Caruso

**Affiliations:** 1 Paul Scherrer Institute (PSI), 5232 Villigen PSI, Switzerland; jjh@enusa.es (J.H.); marco.pecchia@psi.ch (M.P.); dimitri-alexandre.rochman@psi.ch (D.R.); hakim.ferroukhi@psi.ch (H.F.); 2 National Cooperative for the Disposal of Radioactive Waste (NAGRA), 5430 Wettingen, Switzerland; stefano.caruso@nagra.ch

**Keywords:** spent nuclear fuel, geological repository, criticality safety, burnup credit, loading curves

## Abstract

This paper presents preliminary criticality safety assessments performed by the Paul Scherrer Institute (PSI) in cooperation with the Swiss National Cooperative for the Disposal of Radioactive Waste (Nagra) for spent nuclear fuel disposal canisters loaded with Swiss Pressurized Water Reactor (PWR) UO_2_ spent fuel assemblies. The burnup credit application is examined with respect to both existing concepts: taking into account actinides only and taking into account actinides plus fission products. The criticality safety calculations are integrated with uncertainty quantifications that are as detailed as possible, accounting for the uncertainties in the nuclear data used, fuel assembly and disposal canister design parameters and operating conditions, as well as the radiation-induced changes in the fuel assembly geometry. Furthermore, the most penalising axial and radial burnup profiles and the most reactive fuel loading configuration for the canisters were taken into account accordingly. The results of the study are presented with the help of loading curves showing what minimum average fuel assembly burnup is required for the given initial fuel enrichment of fresh fuel assemblies to ensure that the effective neutron multiplication factor, $k_{eff}$, of the canister would comply with the imposed criticality safety criterion.

## 1. Introduction

The Swiss National Cooperative for the Disposal of Radioactive Waste (Nagra) plans to submit a general licence application for a deep geological repository for the disposal of spent fuel and high-level waste (HLW repository) and for low- and intermediate-level waste (L/ILW repository) by 2024. One of the requirements for the design of the HLW repository is the safety of the installations (encapsulation facility and repository) from the point of view of a possible criticality excursion over a 1,000,000-year lifetime. Were it to occur, criticality would affect the properties of the engineered barrier system, namely the canister, and the backfill material and the near-field of the host rock.

For the above reasons, the criticality safety issue for the disposal of spent fuel canisters was preliminarily investigated by Nagra already in 2002 in the context of the safety assessment of a repository for spent fuel and high-level waste in the Opalinus Clay [1]. The results of that study were not considered sufficiently developed for the detailed design of canisters and for a systematic and comprehensive application of burnup credit (BUC) to all Swiss spent nuclear fuel (SNF) assemblies. However, the project came to the important conclusion that a combination of burnup credit and canister design modifications could ensure subcriticality in all cases. Furthermore, the application of BUC was identified as necessary only for the case of PWR SNF but not for the case of Boiling Water Reactor (BWR) [2]. In fact, such findings were basically in line with the studies performed in the USA in relation to the Yucca Mountain deep geologic repository project, as can be seen in Reference [3]. Therefore, a detailed criticality safety analysis for BWR fuel was not considered in the follow-up PSI/Nagra collaboration; however, internal Nagra activities were carried out to demonstrate compliance with the subcriticality criteria without the application of a burnup-credit approach, namely by considering ideal fresh (un-irradiated) fuel, and without any credit from burnable neutron poisons (e.g., gadolinium). Also, the case of a degraded canister/fuel configuration was outside the scope of the project phase described in this paper. This is a certain limitation of the preliminary results presented here since the effects on the criticality evaluations of such medium to very long-term processes as materials corrosion, alteration and dissolution of the fuel matrix [4], as well as canister deformation or even potential geochemical separation of Plutonium [5] have not been yet considered. Such studies are thus planned for investigations in subsequent phases of the PSI/Nagra collaboration.

At the present stage, a dedicated calculation methodology for criticality safety evaluations (CSE) related to interim dry storage and long-term waste disposal is under development at PSI. The CSE+BUC assessments require two coupled calculations: first, fuel depletion and decay calculations to obtain the isotopic compositions of the fuel after the discharge and cooling period and then, criticality calculations using these compositions, for different initial enrichments and final burnups in order to create loading curves. All the relevant details of the calculation methodology applied and of the studies performed are reported below. The numerous validation studies that supported the described methodology development and qualification are not presented here in detail as they have been the subject of many previous publications. In particular, the validation studies for the fuel depletion calculations with the stand-alone CASMO code [6,7] and with the CASMO/SIMULATE codes using PSI proprietary post-irradiation examination (PIE) data [8], for the reactor core-follow simulations with the CASMO/SIMULATE codes using the in-core reactor measurements [9] and for the criticality calculations with the Monte Carlo N-Particle^®^ MCNP^®^ or MCNPX Software (The registered trademarks are owned by Triad National Security, LLC, manager and operator of Los Alamos National Laboratory (see https://mcnp.lanl.gov/; assessed on 29 January 2019).) using criticality benchmark experiments [10,11] should be mentioned as examples.

The paper is structured as follows. Section 2 presents the approach used for defining the bounding SNF case, including the description of the employed tools and models, as well as the criticality safety criterion selected for the bounding case assessments. Section 3 presents and discusses the results obtained for the bounding SNF case definition. Section 4 describes the methodology applied for the loading curve derivation. The integration of the most penalising burnup profiles and random uncertainty components in the CSE+BUC are demonstrated in Section 5. The loading curves for the disposal canisters are finally derived in Section 6. These loading curves are preliminary, since there is still room for improvement, in particular, in relation to the treatment of uncertainties, as discussed later. Furthermore, the effect of degraded canister/fuel configurations on criticality has to be considered in future work for the development of the final loading curves. However, at the same time, these preliminary loading curves can be used to guide future advanced studies and to serve for verification of the updated results. Section 7 and Section 8 provide a discussion of the results and the conclusions on the work performed, respectively.

## 2. Definition of the Bounding SNF Case for Loading in Disposal Canisters 

For the specific case in hand in this paper, namely the application of burnup credit to the long‑term disposal of PWR spent nuclear fuel, this work was focused on the simulation of the Nagra conceptual canister model [12] loaded with the fuel assemblies (FAs) corresponding to the Gösgen nuclear power plant (KKG) fuel designs. To be on a conservative side, optimum moderation conditions must be assumed in criticality safety assessments. The determination of the most reactive moderation was done in the previous preliminary assessments [1] for canisters loaded with PWR/BWR UO_2_ or UO_2_/MOX (5% Pu-fiss) fuels when $k_{eff}$ was calculated as a function of the water density. It was found in those studies that the maximum water density corresponds to the highest $k_{eff}$ values. Thus, in the given work, the loaded canister is also assumed to be flooded with water entering through a postulated breach, as is the case for the Swedish design and related criticality assessment [13]. Additional verification of the optimal moderation conditions will be performed at future stages of the work when the canister design will have been fixed.

The determination of final loading curves for SNF to be loaded in disposal canisters was preceded by a preliminary study, namely the evaluation of the bounding fuel type and/or conservative conditions, which are discussed in the given chapter. The CSEs were realised for PWR FAs using realistic irradiation conditions for different enrichments, burnup levels, FA designs and fuel compositions. Note that the KKG nuclear power plant (NPP) has used the highest possible enrichment of fuel as compared with two other Swiss PWR reactors Beznau 1 and 2 (KKB1 and KKB2). 

### 2.1. Calculation Tools

For the depletion phase, the two-dimensional (2-D) fuel assembly depletion code CASMO5 [14] was employed with feedback from the three-dimensional (3-D) reactor code SIMULATE-3 [15] using the in-house BOHR tool [8,16], as illustrated later in Section 2.3. 

Between the depletion calculations and the criticality calculations, a decay phase is introduced for the study of the long‑term evolution of the fuel composition. The decay module of the code SERPENT2 [17] has been selected as the most preferable based on the analysis of the available options [18].

For the criticality calculation case, the MCNP6 code [19] has been employed as the most validated among the Monte Carlo codes available at PSI. 

### 2.2. Development of Models

**Figure 1:**
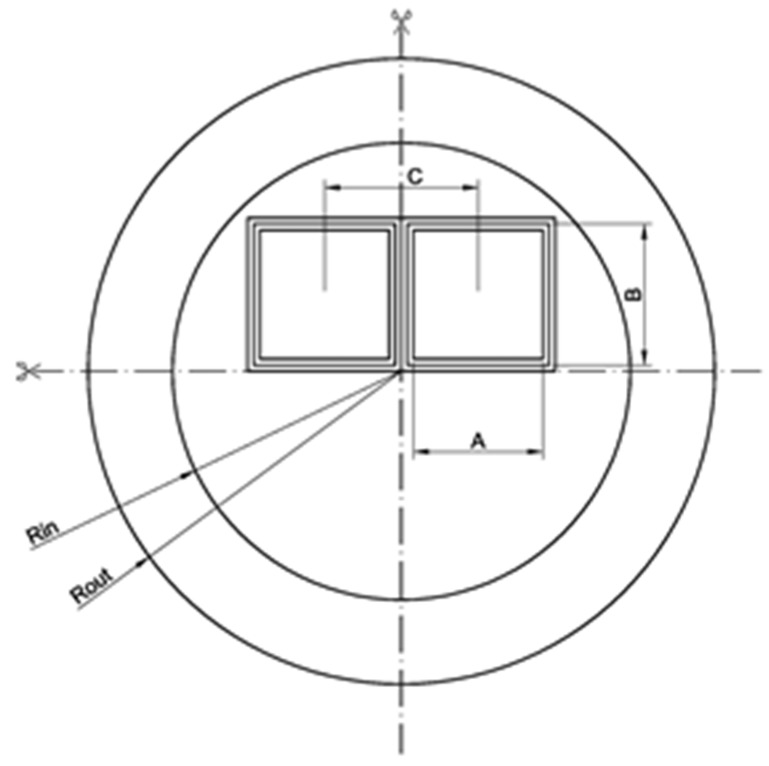
A sketch of the carbon steel disposal canister.

The disposal canister is basically a carbon steel cylinder, is almost 5 metres long and is designed to fit 4 PWR FAs in 4 separate inserted and welded carbon steel boxes (see Figure 1). The preliminary dimensions selected for the present analysis were R_in_ = 41 cm, R_out_ = 55 cm, box centre-centre (C-C) separation = 17.9 cm, A ≈ 21.5 cm (side of the FA top head) and B ≈ 23.5 cm (inner side of the FA box). The analysis performed refers entirely to this disposal canister concept.

No uncertainties related to the underlying nuclear data, depletion calculations and corresponding fuel compositions are considered at this stage, but they will be included later to develop the fuel loading curves. Three types of fuel assemblies were considered for the analysis:

1.  UO_2_ 4.94 w/o U‑235

The UO_2_ assembly is formed by a 15 × 15 array of fuel pins (with 20 guide tubes) which contain fuel homogeneously enriched at 4.94 weight percent (w/o) of U-235 and operated up to 5 cycles, reaching discharge burnups of 17.61, 33.82, 50.47, 61.92 and 72.75 GWd/tHM.

2.  Mixed Oxide Fuel (MOX) 4.80 w/o Pu_fiss_

The MOX fuel assembly contains a distribution of three slightly different enrichments inside the assembly, with the central rod empty, i.e., flooded with water. The content of ^239^Pu and ^241^Pu fissile isotopes in the plutonium fuel fraction is approximately 64%. The chosen assembly was operated to burnups of 18.10, 34.78, 44.96 and 51.72 GWd/tHM.

3.  Enriched Reprocessed Uranium (ERU), 4.599 w/o U-235 Equivalent

The ERU fuel assembly is similar in structure to the UO_2_ fuel assembly and was operated to burnups of 17.27, 34.58, 50.10, 56.04 and 61.72 GWd/tHM. 

The following different loading configurations of the canister were considered for the bounding case analysis:

- 4 similar UO_2_ FAs
- mixed burnup UO_2_ fuel (3 FAs with the same burnup + 1 FA with lower burnup)
- 4 similar ERU FAs
- 4 similar MOX FAs
- 1 MOX FA and 3 similar UO_2_ FAs
- 3 similar UO_2_ FAs and an empty position

Configurations with more than 1 MOX FA loaded in a canister are not considered feasible, as their contributions to the total heat load is too high according to the repository constraints based on the Nagra safety assessment. In fact, a heat load of 1.5 KW is considered the maximum for the disposal canister to ensure the functionality of the engineered barrier system (canister-bentonite-Opalinus Clay). The mixed loading of one MOX with 3 UO_2_ is, however, considered possible for the Nagra conceptual design.

The calculational model is bounded by a 35 cm layer of bentonite clay, saturated with water. Vacuum boundary conditions are employed at the outer surface of the bentonite clay. However, the impact of the bentonite on the system $k_{eff}$ is negligible for a flooded canister. Other conservative assumptions applied to the model are low material temperature and the presence of water without diluted minerals (these assumptions lead to better neutron moderation and less neutron absorption, both leading to an increasing $k_{eff}$ value).

The study was performed with representative assemblies, selected arbitrarily from each assembly batch considered among the assemblies reaching the highest burnups at the End of Life. The irradiation history was reconstructed using real plant operating data, as was described above, with the help of the BOHR tool. Therefore, the best estimate axial burnup profiles were utilised for the bounding fuel case analysis. In this sense, the bounding assessments performed, described in the next section, are based on the best estimate modelling of the fuel operating history.

**Figure 2:**
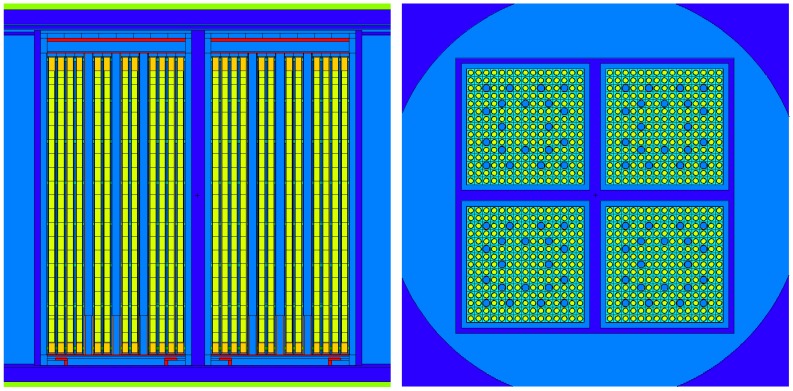
The axial (**left**) and radial (**right**) views of the canister MCNP model loaded with fresh UO_2_ fuel.

Figure 2 shows the MCNP model describing the canister (not in full detail as compared with Figure 6 given later; the model’s outer boundaries are cut as they are not significant for presentation) and the fuel assemblies with the discrete axial spent fuel specifications (see further details in Section 4.2.1). 

### 2.3. The Calculation Route

The computational scheme developed and implemented at PSI [16] is based on the suite of reference CASMO5/SIMULATE-3 core models, continuously developed and validated for all Swiss reactors and all operated cycles within the PSI Core Management System (CMSYS) platform [20]. These core models are based on the real reactor operating histories. The core nodal-wise thermal-hydraulic conditions are calculated with the SIMULATE-3 code using the plant-provided boundary conditions on the inlet coolant temperature and core exit coolant pressure during the reactor cycle.

**Figure 3:**
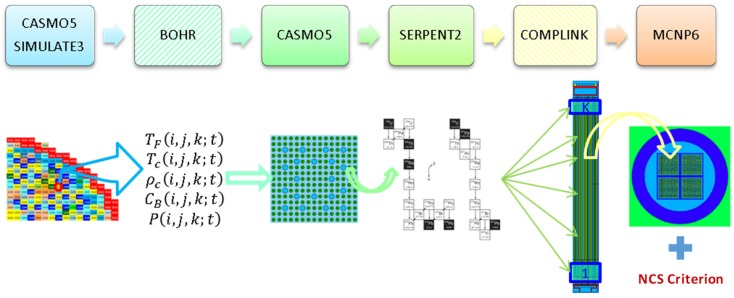
The computational scheme for the burnup credit in a disposal application.

A principal limitation of the SIMULATE-3 code is that it is not suited for providing detailed spent fuel composition (SFC), which is required for the criticality calculations, because such data are not needed for the two-group nodal diffusion approximation employed in SIMULATE-3 for full core neutron transport calculations. The detailed fuel isotopic composition is actually required at the stage of preparation of the neutron cross section libraries with the CASMO5 code, which involves a higher order method of characteristics for the neutron transport solution at the spatial level of the FA axial slice. To overcome the limitations of the presently used CASMO5/SIMULATE-3 system of codes with respect to the BUC application calculation needs for which this system of codes was not designed, a specific calculation scheme was developed called BOHR (which stands for “Bundle Operating History Reconstruction”). BOHR can provide CASMO5 with the realistic operating history conditions, which can be retrieved from SIMULATE-3 full core calculations, to rerun CASMO5 depletion calculations and obtain the pin‑by‑pin detailed burnt fuel isotopic composition for an axial slice of the fuel assembly. Thus, CASMO5 depletion calculations are performed up to the “discharge burnup” for each particular axial FA slice, with the actual operating history which is known from the SIMULATE-3 core-follow calculations (when conservative axial burnup profiles are applied, the burnup does not correspond to realistic discharged burnup but is defined based on the conservative assumptions, as explained later in Section 4.2.1). Thus, the power history in the CASMO calculations following BOHR procedures (third step in Figure 3) is exactly the same as was used in the original SIMULATE calculations, i.e., detailed (e.g., daily-wise) power history provided by the plant operator.

In the next step, the burnt compositions are translated into the input file of SERPENT2 to compute the change in the isotopic concentrations during different decay periods. In other words, the fuel depletion is simulated in the given approach with the CASMO code, while discharged spent fuel decay is done with SERPENT (recall Section 2.1). The coupling between the basic MCNP canister model and the CASMO5/SERPENT results is performed with the help of the COMPLINK tool [21], which imports detailed SNF compositions for every assembly to define a complete canister loading.

The MCNP model of the canister loaded with burnt fuel assemblies is then used to compute the $k_{eff}$ of the system at different time steps during the decay, aiming to assess if the system remains subcritical in the postulated case of a flooded canister. In order to ensure criticality safety, the calculated $k_{eff}$ must be assessed with the Nuclear Criticality Safety (NCS) Criterion. Figure 3 provides an illustration of the scheme and related processes described in the following subsections. Here, the symbols *T_f_, T_c_,* *r_c_, C_B_* and *P* mean respectively the fuel temperature, the coolant temperature, the coolant density, the soluble boron concentration in the coolant and the coolant pressure in the computation node (*i,j,k*) (the axial slice of a fuel assembly in the SIMULATE-3 full core 3-D model) in the x,y,z system of coordinates at time *t*.

#### 2.3.1. Retrieval of the Nodal History

The sequence of calculations begins with the generation of the data library using CASMO5 2D lattice calculations. The nuclear data library employed in this step was based on the ENDF/B-VII.0 library [22]. The spacers are smeared along the full axial length of the FA, which complies with the SIMULATE‑3 model requirements. Every FA type is computed, and the initial isotopic composition is taken from the final state of the previous cycle.

The reactor cycle operation is computed with SIMULATE-3. From these results, the values for the state parameters are retrieved for every FA and axial elevation at different cycle instants. The explicit spacer model for the neutronics solution is activated in SIMULATE-3.

The BOHR tool is employed for the extraction of the required values from SIMULATE-3, which provides the values for the nodal power, the fuel temperature, the coolant temperature and density and the boron concentration for every axial and radial location in the nodal calculation. The presence of inserted control rods during the operation is also taken into account.

#### 2.3.2. Lattice Calculations for Discharge Composition Estimation

In order to extract and make use of the composition for every fuel pin at the discharge burnups and for every axial FA slice, new 2-D lattice calculations are performed with CASMO5. During every time interval, the actual irradiation history (fuel and coolant temperatures and densities, positions of control rods, etc.) is employed based on the core cycle depletion/operation calculations with SIMULATE-3. The nuclear data library used at this stage was upgraded to ENDF/B-VII.1 (The new release of the nuclear data library for CASMO5 became available at the time of the study and was therefore employed for the new calculations; however, the original CASMO5/SIMULATE-3 models described in the previous section were based on the older ENDF/B-VII.0 version. Nevertheless, this inconsistency should not be of any practical significance for the present analysis.) [23]. 

The spacer mass is, again, smeared along the whole axial length, introducing an approximation to realistic conditions. The additional approximations are that the irradiation-induced changes of the FA structures and the materials are currently not taken into account. More importantly, it must also be underlined that the CASMO5 reconstructed depletion calculations are performed using single assembly reflected models, i.e., without accounting for a realistic leakage term representative of the 3-D environment under which the assembly was irradiated. The impact of this approximation and the ways to improve it still need to be analysed. It should be noticed, however, that the effects of the intra-fuel assembly pin-by-pin horizontal burnup distributions are taken into account later at the stage of the full-scale MCNP criticality calculations for the loaded canister, as presented in Section 4.2.2 and Section 5.2. It is foreseen that the application of the newer Studsvik codes SIMULATE5 and SNF may allow for the improvement of the presently developed BUCSS-R methodology (see later Section 7.1). 

#### 2.3.3. Decay Calculations after Discharge

From the detailed burnup results, the compositions obtained are decayed over a one-million-year period using the Transmutation Trajectory Analysis algorithm programmed in the burnup module of the code SERPENT2 [17]. The decay data from ENDF/B-VII.1 were employed. The concentrations were computed at the following times: 0, 1, 2, 5, 10, 20, 40, 60, 80, 100, 120, 150, 200, 300, 500, 1000, 2000, 5000, 8000, 10,000, 15,000, 20,000, 25,000, 30,000, 40,000, 45,000, 50,000, 100,000, 500,000 and 1,000,000 years. The fuel pin compositions in the FA decay individually at every axial elevation.

#### 2.3.4. Criticality Calculations for the Disposal Canister

Each particular MCNP model, which includes the SFC for every discharge burnup at the end of each fuel operation cycle and for each decay period, is generated starting from a base input file with the canister model loaded with FAs of equal uniform composition corresponding to fresh fuel (illustrated in Figure 2). The tool COMPLINK is used for this purpose. The SFC is provided at the pin-by-pin and axial node-wise level, as further explained in Section 4.2.1.

Only the isotopes usually accounted for in burnup credit calculations are included in the fuel composition. Two sets of isotopes are considered following the specifications given in Reference [24], customarily termed the actinides only (AC) and the actinides plus fission products (AC+FP) groups. The AC group includes U-233, U-234, U-235, U-236, U-238, Pu-238, Pu-239, Pu-240, Pu-241, Pu-242, Am-241, Cm-242, Cm-243, Cm-244, Cm-245 and Cm-246, and the AC+FP group includes in addition to the isotopes in the AC group Np‑237, Am-242^m^, Am-243, Mo-95, Tc-99, Ru-101, Rh-103, Ag-109, Cs-133, Nd-143, Nd-145, Sm-147, Sm-149, Sm-150, Sm-151, Sm-152, Eu-151, Eu-153 and Gd-155. Note that compared with the list of isotopes considered in Reference [24], here, the curium isotopes are also taken into account.

The nuclear data from ENDF/B-VII.1 coming with the MCNP6 software distribution have been used in the calculations. The canister and fuel geometry are considered to be constant over time, which is a common approach in this type of preliminary criticality safety assessments [13]. The material temperature is 293.16 K everywhere, and corresponding densities and dimensions are employed. 

The MCNP criticality calculations for most of the analysed cases were performed with 80 inactive cycles and 60 active cycles. The number of inactive cycles was chosen following the MCNP recommendation based on the Shannon entropy estimation of the fission distribution [25] for the AC+FP case with mixed MOX and UO_2_ fuels, as this is the most challenging in terms of initial fission source convergence. All the cycles were run with 200,000 neutron histories each. The resulting $k_{eff}$ statistical uncertainty ($k_{eff}$ standard deviation reported by the MCNP code) was approximately ±25 pcm.

### 2.4. Criticality Criterion Selected for the Bounding Case Assessments

The criterion for nuclear criticality safety assessment, conventionally termed the Upper Subcritical Limit (USL) hereafter, which accounts for the burnup credit of the spent nuclear fuel, is discussed in detail later in Section 4.1. Here, it must only be mentioned that the criticality safety criterion may vary depending on the methodology definition and on the national regulatory requirements. However, one particular component is basically common to all countries and methodologies, namely the so-called “administrative margin” (also referred to as “an arbitrary margin to ensure the subcriticality” [26]), which will be denoted here as $\Delta k_{eff}^{AM}$. Normally this margin equals 0.05 in terms of the absolute $k_{eff}$ value [13,27], and it is typically dominant compared to all other margins and imposed uncertainty components. The last comment is especially true for the fresh fuel case, but even in the case of spent fuel, the depletion-related uncertainties and margins, e.g., assessed in Reference [13], are noticeably smaller as compared with 0.05. Thus, the USL values obtained with different methodologies by different organisations and countries for the same type of applications may vary but are normally not too far from the $k_{eff}$ = 0.95 value. Therefore, for the sake of a better representability and an easier comparison with similar studies, it makes sense to consider the $k_{eff}\;$= 0.95 value as the simplified criticality criterion selected for the consequent bounding case assessments, while for the final loading curve derivations, the PSI methodology-specific USL value will be employed.

## 3. Results of the Bounding Fuel Case Assessments

### 3.1. Fresh Fuel

To start, a set of calculations has been performed with fresh fuel configurations. The configurations considered are the following:

- The canister in dry conditions (actually filled with helium gas)
- The canister flooded with water at 293.16 K
- The canister flooded with the FAs displaced diagonally towards the centre of every fuel box (within the design tolerances)
- The canister flooded with the FAs displaced diagonally towards the outer part of the fuel box

When several assemblies of the same type are considered (UO_2_, ERU and MOX), they are assumed to be fully identical (originating from the same batch, i.e., with the same nuclear and mechanical design). The calculated $k_{eff}$ values for each of these cases are presented in Table 1. 

The canister in dry conditions is clearly subcritical for any combination of fresh fuel. However, in all other cases (except the case of MOX/flooded outwards), the calculated $k_{eff}$ values are above 0.95 and the $k_{eff}$ increases notably if a less favourable position of the assemblies inside the canister is considered (inwards). The importance of the distance between assemblies for the $k_{eff}$ is clearly reflected in the table. This also means that the material of the wall boxes has a small impact on decoupling the neutron fluxes of each assembly, so the loading position of the assemblies into the canister seems to be important for the $k_{eff}$ values in flooded conditions.

### 3.2. Discharged Fuel

The burnup credit approach can reduce the system $k_{eff}$ (In fact, the major change in the system $k_{eff}$ which occurs due to taking into account the burned fuel composition is associated with the change in the infinite multiplication factor of the fuel lattice (*k*_∞_), while the changes in the neutron leakage should be the effects of the second order. Nevertheless, for the sake of rigor, all presented evaluations were done for realistic 3-D canister models, and therefore, only the results for the system $k_{eff}$ are discussed in this work.) as a consequence of the presence of neutronic poisons (fission products and some non-fissile actinides), as well as due to the depletion of multiplicative materials (major actinides) (Although some amount of fissile major and minor actinides is produced in the reactor operation, the net $k_{eff}$ (primary *k*∞) effect with fuel burnup is negative.). The impact on $k_{eff}$ has been evaluated for both the AC and AC+FP cases.

For these calculations, the same configurations (as the fresh fuel case) are used; however, in this case, the canister is loaded with an SFC with different burnups and at different cooling times, covering the one-million-year period.

**Figure 4:**
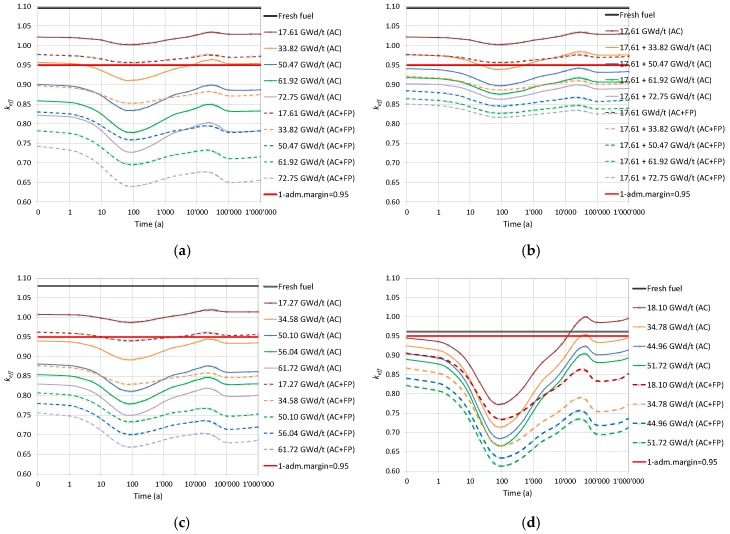

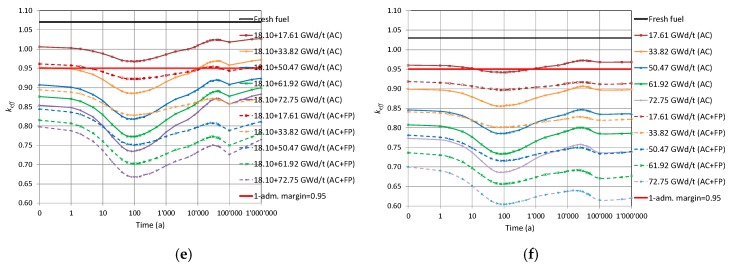
The evolution of $k_{eff}$ for the intact canister loaded with (**a**) spent UO_2_ fuel; (**b**) mixed burnup UO_2_ fuel (the first burnup value for one position, the second value for the three remaining); (**c**) ERU fuel; (**d**) MOX fuel; (**e**) one low burnup MOX (18 GWd/tHM) and three UO_2_ fuel at different burnups; and (**f**) 3 UO_2_ assemblies and one empty position.

For the burnt fuel configurations, the analyses were conducted for each of the assembly-averaged burnup levels reached after every reactor cycle. The spent fuel compositions keep changing after irradiation due to the decay processes, and therefore, the fuel neutronic properties (“reactivity”) are changing as well. The calculated curves of the $k_{eff}$ evolution are plotted in Figure 4 for a flooded canister at different instants during the decay of the isotopes for both AC and AC+FP cases. 

The results obtained are summarised below in Section 3.2.1, Section 3.2.2, Section 3.2.3 and Section 3.2.4. It should be mentioned that the observed behaviour is in line with already published results for similar situations analysed and discussed in detail in Reference [28]. In brief, it can be recalled here that the decrease of $k_{eff}$ within the first approximate 100 years is mainly related to the decay of ^241^Pu (T_1/2_ ≈ 14.4 years), as well as the build-up of ^241^Am (in the case of AC+FP, also the build-up of ^155^Gd as a result of the beta-decay of ^155^Eu is important). Later, the increase of $k_{eff}$ up to around 30,000 years is explained by the decay of ^241^Am (T_1/2_ ≈ 432 years) and ^240^Pu (T_1/2_ ≈ 6,560 years). At a later time, $k_{eff}$ starts to decrease again mainly due to ^239^Pu decay (T_1/2_ ≈ 24,100 years). The given explanations and the half-life (T_1/2_) data are borrowed from Reference [28].

#### 3.2.1. UO_2_ Fuel Assemblies

Figure 4a shows the $k_{eff}$ results as a function of the decay time obtained for the disposal canister loaded with the UO_2_ FAs of the same burnup for both the AC and the AC+FP approaches. It was assessed using quadratic interpolation that the spent fuel with a burnup of less than 24 GWd/tHM (For the bounding fuel case assessments reported in this section, a quadratic interpolation technique was applied to estimate the limiting burnup values based on the data illustrated in Figure 4. A more robust method explained in Section 6 was finally applied for the derivation of the limiting burnup values for the loading curves.) (for the AC+FP case) does not meet even the limit of $k_{eff}$ = 0.95 for loading unless a mixed configuration with higher burnup fuel was to be considered. If an AC approach was considered, a burnup higher than approximately 38 GWd/tHM needs to be reached. The $k_{eff}$ of the system after 10,000 years could reach a value above that of the initial discharged fuel for the AC approach. 

#### 3.2.2. UO_2_ Fuel Assemblies of Mixed Burnups

Next, a mixed burnup configuration was investigated, as reported in Figure 4b. One FA at low burnup (17.61 GWd/tHM) is considered in the mixed loading with the other three FAs of higher burnups. The results show that high burnup fuels are needed (above 50 GWd/tHM) when taking credit only for actinides.

#### 3.2.3. ERU Fuel Assemblies

The ERU FA corresponds to fuel enriched up to 4.6 w/o ^235^U_eq_ and operated until 61.72 GWd/tHM. The evolution of $k_{eff}$ through time, shown in Figure 4c for both the AC and AC+FP cases, is very similar to that of UO_2_ fuel. 

#### 3.2.4. MOX Fuel Assemblies

The MOX FA corresponds to fuel enriched to 4.8 w/o Pu_fiss_ and operated until 51.72 GWd/tHM. The evolution of $k_{eff}$ through time in Figure 4d has stronger dip and peak values around 100 and 30,000 years (case AC+FP) or 45,000 years (case AC), respectively. Notable is that the $k_{eff}$ peak after thousands of years would be higher than any previous value calculated for the AC case, meaning that using the discharge compositions without decay would not be a bounding assumption for the whole disposal period (moreover, taking only actinide changes into account leads to a remarkable case where $k_{eff}$ of partly burned fuel can go above the $k_{eff}$ of fresh fuel; see the Figure 4d case for 18.1 GWd/tHM (AC)).

On the other hand, considering additional minor actinides and fission products in the fuel composition also has a stronger impact on the $k_{eff}$ than for UO_2_ fuel, and this impact is more important at later periods, thus reducing the $k_{eff}$ peak below former values in time. Therefore, the use of the discharge compositions would be bounding for the AC+FP approach. 

#### 3.2.5. One MOX and Three UO_2_ Fuel Assemblies 

In this model, the canister is loaded with one MOX and three UO_2_ FAs. Figure 4e shows the $k_{eff}$ evolution for the canister loaded with low burnup MOX (18 GWd/tHM) together with the three UO_2_ assemblies at different burnup levels. The main findings from these graphs are as follows:

- For the AC case, $k_{eff}$ is increasing from 100,000 years and keeps growing at the end of the period considered of 1 million years. At the cooling time of 1 million years, the $k_{eff}$ values are greater than at the zero cooling time.
- For the AC+FP case, the bounding value of $k_{eff}$ corresponds to zero cooling time.

The AC approach would require UO_2_ fuel burnt to around 40 GWd/tHM, and the AC+FP approach would require burnup of approximately 20 GWd/tHM.

#### 3.2.6. Empty Position and Three UO_2_ Fuel Assemblies

The empty position is assumed to be also flooded with water. The results plotted in Figure 4f show that fuel with a limiting burnup of 19 GWd/tHM can be considered if using the AC approach and 10 GWd/tHM for the AC+FP approach. 

### 3.3. Findings from the Bounding Fuel Case Analysis

The bounding fuel case analyses consisted of assessing the multiplication factor variation as a function of the discharge burnup and decay time, ranging between fresh fuel conditions and best-estimate burnt fuel configurations. The main findings of the analysis are that UO_2_ fuel could be problematic if featuring low burnups, especially for the AC case where all fuels suffer a rise in $k_{eff}$ in later time periods after disposal, which may violate the USL value. ERU fuel has a similar behaviour to the UO_2_ fuel. The mixing of UO_2_ and MOX fuel in the canisters could be a good compromise to keep $k_{eff}$ below the safety margin while avoiding the thermal limitations more easily violated by MOX fuel filled canisters.

**Figure 5:**
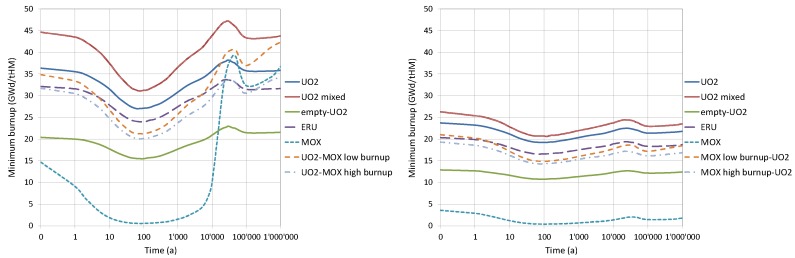
The evolution of minimum burnup credit required to comply with a $k_{eff}$ value below 0.95 within the geological disposal timeframe: AC case (**left**) and AC+FP case (**right**).

Figure 5 summarises the minimum burnup credit which would be required for every type of loaded nuclear fuel considered in this study for both the AC and AC+FP cases. 

For the AC case, the loadings of UO_2_ fuel operated for just one cycle (17.61 GWd/tHM) could be allowed only if mixed with 3 other UO_2_ assemblies with burnups above 45 GWd/tHM (the second $k_{eff}$ peak is decisive for the AC approach). It can also be noted from Figure 5 for the AC case that the minimum burnup required for a homogeneous UO_2_ fuel loading is not always above the minimum burnup required for the canisters filled with MOX fuel (mixed UO_2_/MOX case or full MOX case) since the second $k_{eff}$ peak in the MOX cases is very high.

For the AC+FP case, the mixed burnup loading (1 MOX + 3 UO_2_) requires a minimum burnup of 20 GWd/tHM for the UO_2_ fuel (fuel operated at least for two operating cycles). In this case, the burnup credit required for canisters loaded with MOX fuel would be dominated by the $k_{eff}$ at discharge and it will not be higher than the corresponding UO_2_ case.

Finally, it should be borne in mind that all these results imply that the fuel matrix is still intact, maintaining the actinide and fission product mixture even after 100,000 years which is far from the normal FA design target, which ensures FA integrity during irradiation in core but not for the geological timeframe.

The main driving parameter for criticality in the current canister design is the distance between the FAs, due to the compact configuration (and also because neutronic poisons are not present in the canister basket material). Fresh fuel calculations indicate a difference of 2–5% in $k_{eff}$ values between nominal and displaced configurations, which is very important.

The results obtained are in line with the analyses performed for other canister designs [28] and indicate that the inclusion of the fission products in the burnup credit could allow 2-cycle-operated FAs to be safely loaded into disposal canisters. However, the margin is very close to the limit, and once the biases, uncertainties and further bounding assumptions are introduced for the loading curves generation, it will be further reduced, as will be discussed in the following sections.

Fuel with low burnup (corresponding to one cycle of operation) cannot be loaded to fill all the positions of the same canister. A dedicated study with mixed UO_2_-MOX models demonstrated that the fuel with low burnup could meet the requirements in some mixed configurations or in the empty position configuration.

Finally, based on the reported findings for the currently considered canister design, the canister model loaded with 4 similar PWR UO_2_ spent FAs (i.e., the type of fuel employed at KKG) will be used for the preliminary loading curve derivation as it is the most problematic configuration among those studied.

## 4. Methodology for Preliminary Loading Curve Derivation

As a main outcome of the study outlined to this point, the application of burnup credit to the criticality calculations for disposal canisters appears to be necessary for the safe disposal of the PWR spent FAs operated in the Swiss reactors according to the current design concept for the Nagra disposal canister.

This section presents the results of the final stage of the preliminary loading curve derivation. The application of a best-estimate computational route is now complemented with conservative coverage in the form of a stochastic analysis of the main uncertainty components in combination with the application of bounding burnup profiles, as illustrated below. Furthermore, the USL value considered in this final stage of the work is now based on the comprehensive validation study for LWR fuel performed at PSI for the MCNP code in combination with the ENDF/B-VII.1 library, which has been selected for the routine criticality calculations [10,11]. 

Note that the general practice in the burnup credit applications is based on choosing a set of bounding parameters for the burnup calculations in terms of power density, fuel and coolant temperatures, coolant densities, etc. so that the $k_{eff}$ at discharge for such conservative assumptions will be higher than the $k_{eff}$ obtained with any possible real irradiation history. This path, however, was not adopted for the BUCSS-R project. In fact, the approach in the BUCSS-R project is different because real operational data are employed (using SIMULATE-3 for accurate core-follow calculations) for the FAs of different enrichments in order to estimate the loading curves on the basis of best-estimate assessments integrated with a conservative but rational treatment of the uncertainties. Therefore, at present, only some representative FAs operated at KKG could be considered and explicitly analysed. It is foreseen that, in the future, the studies performed should be repeated for a statistically significant number of FAs, at least for the most reactive design/enrichment types, to allow for statistically confident verification/updating of the presently evaluated preliminary loading curves.

### 4.1. Chosen Criterion for Criticality Safety Accounting for Burnup Credit

The criticality safety criteria applied for derivation of the loading curves can be presented with the following relations (adapted from Reference [29]):

(1) $${k_{eff}|}_{Bounding\;FA\;pos}^{Canister}\left(BU\right)+\Delta k_{eff}^{Ax}\left(BU\right)+\Delta k_{eff}^{Rad}\left(BU\right)+2\sigma_{tot}\left(BU\right)<USL={K_{eff}^{LTB}|}_{AOA}-\Delta k_{eff}^{AM},$$

where

(2) $$\sigma_{tot\;}^{}\left(BU\right)=\sqrt{\sigma_{ND}^{2}{\left(BU\right)}^{}+\sigma_{BU-eff}^{2}{\left(BU\right)}^{}+\sigma_{OC}^{2}{\left(BU\right)}^{}+\sigma_{TP}^{2}+\sigma_{T1/2}^{2}+\sigma_{MC}^{2}};$$

(3) $$\sigma_{ND}\left(BU\right)=\sigma_{ND-SNF}^{CASMO}\left(BU\right)+\sigma_{ND-Keff}^{MCNP}\left(BU\right);$$

${k_{eff}|}_{Bounding\;FA\;pos}^{Canister}$ is the neutron multiplication factor corresponding to the disposal canister loaded with SNF placed in the most penalising positions considering the canister technological tolerances (see Section 3.1 for details);$\Delta k_{eff}^{Ax}$ and $\Delta k_{eff}^{Rad}$ are the $k_{eff}$ penalties to cover the bounding axial and radial burnup profiles, respectively; USL is the Upper Subcritical Limit (see Reference [10] for details); and $\sigma_{ND},\;\sigma_{BU-eff}^{},\sigma_{OP}^{},\;\sigma_{TP}^{},\;\sigma_{T1/2}^{}$ and $\sigma_{MC}^{}$ are the uncertainties at one standard deviation level respectively for the nuclear data (*ND*), radiation/burnup-induced changes/effects (*BU-eff*), operating conditions (*OC*), technological parameters components (*TP*), decay constants (*T_1/2_*) and the Monte Carlo statistical uncertainty of the employed MCNP code for the criticality calculations. The listed components of the $\sigma_{tot\;}^{}\left(BU\right)$ uncertainty are assumed to be random (not systematic) and uncorrelated. The resulting $\sigma_{tot\;}^{}\left(BU\right)$ is further assumed to be normally distributed. Under these conditions, the term $2\sigma_{tot\;}^{}\left(BU\right)$ in Equation (1) is assumed to represent the 95% confidence interval for $k_{eff}$, which is, for instance, in line with the recommendations provided, e.g., in References [30,31].

The $\sigma_{ND-SNF}^{CASMO}$ nuclear data-related component is responsible for the $k_{eff}$ uncertainty associated with the SFC (due to the propagation of nuclear data uncertainties during depletion calculations), and the $\sigma_{ND-Keff}^{MCNP}$ component is the $k_{eff}$ uncertainty due to the nuclear data uncertainties themselves (see Section 5.3.3. for further details). 

The ${K_{eff}^{LTB}|}_{AOA}$ term stands for the Lower Tolerance Bound for the particular Area of Applicability (AOA; here, this is limited to Swiss LWR fuel), and its value was reported in Reference [10] as 0.99339 (following the Gaussian-based derivation; see Reference [10] for details) for the PSI CSE methodology using the MCNP code in conjunction with the ENDF/B-VII.1 library. As outlined in Section 2.4, $\Delta k_{eff}^{AM}$ is the “administrative margin” normally imposed to cover the unknown uncertainties to ensure subcriticality, which is assumed here to be 0.05000 (The administrative margin to criticality is normally set at 5000 pcm, i.e., the *k_eff_* of the system plus the calculation bias and uncertainty in the bias should not exceed 0.95. More recently, an administrative margin of 2000 pcm was suggested for very unlikely accident conditions [32].) (5000 pcm). Thus, for the final loading curve derivations, USL = 0.99339 − 0.05000 = 0.94339 is employed. As for the above listed components of the $\sigma_{tot}^{}$ uncertainty, they are presented in more detail in Section 5.4. 

### 4.2. Spatial Burnup Distribution Assumptions

This section comprises most of the modelling and simulation assumptions employed. First of all, it addresses the derivation of bounding burnup profiles and the assessment of their impact on the canister $k_{eff}$ value, to derive the $\Delta k_{eff}^{Ax}$ and $\Delta k_{eff}^{Rad}$ penalties for Equation (1). The impact of the cooling time is also addressed.

#### 4.2.1. Axial Burnup Distribution

Axial burnup profiles of the spent fuel operated at KKG and irradiated to different average burnups were retrieved from the PSI CMSYS database, which includes all the burnup values per fuel assembly at every axial node of the SIMULATE-3 calculations, as described in Section 2. The burnup profiles were normalised with these average values and separated into two families corresponding to the models with 40 axial nodes in SIMULATE-3 with active fuel regions between 358 and 352 cm height and the models with 38 axial nodes in SIMULATE-3 with an active region of 340 cm height. The reason for having SIMULATE-3 models with different numbers of axial layers of nodes with fuel is that the active fuel length of KKG FAs was changed from earlier cycles to the later ones. The older FAs have reflector segments at the 2 bottom nodes instead of fuel nodes. It can also be noted that the older and shorter FAs had lower fuel enrichment compared to the later and longer FAs. Therefore, it is important to take into account an actual axial burnup profile for every specific enrichment for the correct derivation of the loading curves.

**Figure 6:**
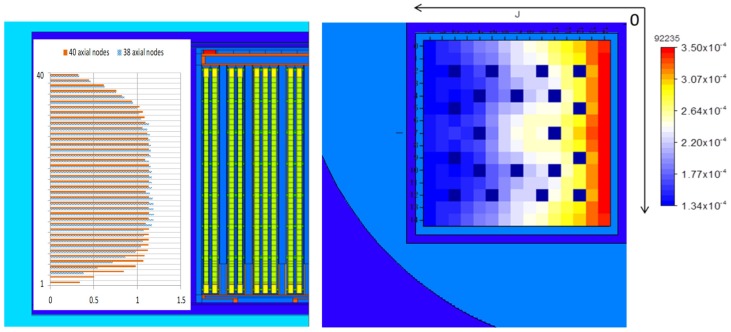
The illustrative MCNP model (1/4th symmetry sector; schematic and not to scale): the axial (**left**) and radial (**right**) view of the refined canister model. The bounding axial burnup profiles (relative units) for the models with 38 and 40 axial fuel nodes and radial burnup profiles (here, the U‑235 atomic density is *10^24^at/cm^3^) are illustrated.

Following standard practice [33], the approach was to choose the lowest normalised burnup values of all the profiles for the first and the last 9 nodes and the highest normalised burnup values of the profiles for the remaining central nodes. This resulted in the renormalised profiles shown later in Figure 6 (the average node burnup fraction is normalised to unity in both the cases of 38 and 40 axial nodes).

The change in the bounding axial profiles with the average assembly burnup was not considered and could be a way of reducing conservatism if needed. Also, a mass calculation with all the profiles in the database could be a different approach to deriving bounding profiles.

#### 4.2.2. Radial Burnup Distribution

For the radial burnup profiles within the FAs, there were no operational or CMSYS data available at the time of the study (such data could be obtained by upgrading the reference CMSYS models and also can be obtained in line with the discussions provided later in Section 7). Therefore, as an alternative solution, the publicly open information on the bounding horizontal burnup profile reported in Reference [34] was employed (and this is one of the items requiring further verification). The bounding profile is expressed with Equations (4) and (5), which were derived from real measurements (see Reference [34] and references therein for details) to generate a radial burnup tilt varying for each assembly row:

(4) $$B_{rel}=\frac{B_{H}-B_{av}}{B_{av}}=0.33-\frac{0.08}{15}\cdot \left(B_{av}-10\right),$$

(5) $$B\left(n\right)=\left[B_{rel}+1-\frac{4}{N}\cdot B_{rel}\cdot \left(n-\frac{N+2}{4}\right)\right]\cdot B_{av},$$

where *N* is the number of rows in the square assembly, 15 in our case; $B_{rel}$ is the relative difference between the horizontally averaged burnup value for the half of the assembly ($B_{H}$) with the highest burnup and the horizontally averaged assembly burnup $B_{av}$; and n is the row number in the assembly to which the computed burnup B(n) corresponds (The second formula includes a correction of a sign from the one in the original report [34].).

It must be underlined that, in fact, CASMO5 does not allow the specification of the burnup value desired for each row of the FA. To overcome this difficulty, a surrogate approach was utilised: the CASMO5 input file was modified such that the fuel composition is printed at the 15 burnup steps which correspond to the desired burnups of each of the fuel rod rows, as obtained with Equations (4) and (5). After this, the fuel compositions from every burnup step were transferred to the SERPENT and later to the MCNP6 models row by row, providing required intra-assembly fuel burnup horizontal distributions. In this sense, the approach differs slightly from the one described in Reference [34], where it was assumed that “all the fuel rods belonging to one and the same row have one and the same burnup”. In the present approach, each fuel rod has its own composition, but the horizontally averaged burnup of the entire row is preserved as defined by the above procedure. However, an examination of the typical ratios between the burnup value of each pin and the average assembly burnup showed that, to avoid burning the pins in the regions of higher power above the desired value for the row, a factor of 0.93 should be applied to each B(n). This implies that the assembly burnup is lowered by 7%, which introduces an additional conservatism in the sequence as peripheral pins typically have already lower burnup than average. In addition, the lowest burnup regions of the assemblies are later faced in the canister so as to produce the highest $k_{eff}$.

To illustrate the outcome of the employed methodology, Figure 6 below shows an example of the radial (horizontal) U-235 concentration distribution on a pin-by-pin basis within a FA axial node. 

### 4.3. Impact of Cooling Time

The cooling time between cycles was explicitly considered in the burnup calculations with CASMO5. As it was illustrated in Section 3, in the case of the actinides only credit, the impact of cooling time after discharge on the system $k_{eff}$ is characterised by an initial decrease in $k_{eff}$ in the first approximate 100 years and then a steady increase which reaches its maximum at around 30,000 years after discharge. The important point is that the $k_{eff}$ value at that time for intact canister configurations could be higher than the initial $k_{eff}$ value just after discharge, so taking this initial value cannot be considered bounding in all cases and that decay calculations up to 100,000 years also need to be considered to generate the loading curves. Beyond that time, the flooded intact canister approximation would be totally unrealistic for a canister with a lifetime of approximately 10,000 years, and degraded models should start to be considered in that range.

The time positions where decay compositions have been used to compute $k_{eff}$ values were 0, 5, 20,000, 30,000, 40,000 and 50,000 years.

### 4.4. Canister Modelling

At the stage of the loading curve analyses, the canister model was updated based on the most detailed and actual design information received by PSI from Nagra. The modelling included all the details provided in Reference [12] as well as the detailed structure of the FAs, including heads, grids and rods from the internal documentation available at PSI. The illustration of the final MCNP model with the indications of the spatial burnup distributions employed in the loading curve derivation analysis is shown in Figure 6. 

As outlined in Section 3, the fuel assemblies were conservatively placed towards the centre of the canister at the storage positions as this was found to be the most reactive configuration.

## 5. Quantification of the Bounding Effects and Random Uncertainties Components

The results for criticality calculations of the canister loaded with the same fuel assembly in the four positions were compiled for the different enrichments covering the values employed from the initial to the latest fuel cycles of KKG. In the following sections, the $\Delta k_{eff}$ effects resulting from substituting the nominal burnup profiles by the penalising (conservative) ones are quantified and reported.

### 5.1. Axial Burnup Effect

The results of substituting the original burnup profiles by the penalising profiles while keeping the average assembly burnup are illustrated in Table 2 for the highest considered fuel enrichment of 4.94 w/o.

At first glance, it can be observed that the impact on $k_{eff}$ is stronger for the following cases:

- AC+FP credit
- Longer decay periods
- Increasing burnup

Table 3 shows similar information for the lower enrichment of 3.5 w/o. In this case, the proposed profile is conservative even for the lowest burnup, so the impact of the conservative axial burnup profile is apparently also stronger with lower enrichments. As in the previous case, the added $k_{eff}$ is notably larger in the AC+FP approach.

Finally, regarding the effect for the lowest enrichments of 1.9 w/o and 2.5 w/o, the impact of the proposed profiles is not conservative and the $k_{eff}$ from the nominal profile is higher and will be maintained for the final loading curves. Other profiles could be proposed to create a unique bounding axial burnup profile for these enrichments.

### 5.2. Radial Burnup Effect

In this section, the impact is considered of the intra-assembly burnup profiles obtained in accordance with the descriptions given in Section 4.2.2, in such a way that the lowest radial burnup regions are facing the centre of the canister to raise $k_{eff}$. Results for the cases of 4.94 w/o and of 3.5 w/o are given respectively in Table 4 and Table 5.

In general, the $k_{eff}$ impact is

- stronger for the AC+FP credit;
- increasing from lower to higher burnups; and
- mainly increasing during the decay period up to 20,000 years and then stabilising.

As with the axial burnup profiles for the lowest enrichments of 1.9 and 2.5 w/o, the proposed radial profiles are not conservative in any case and so the nominal profiles are kept.

### 5.3. Assessment of Uncertainties

In the following subsections, quantitative assessments are given for the uncertainties components of $\sigma_{tot\;}^{}\left(BU\right)$ from Equations (1)–(3) listed in Section 4.1. It must be outlined that, at present, some of the values provided are rather preliminary and may require verification depending on the availability of related information.

#### 5.3.1. Reactor Operating Conditions and Radiation-Induced Changes

The impact of the reactor operational parameter variations was estimated in a dedicated study with the help of the CASMO-5 code (the results have been partly presented in Reference [8]). As mentioned, proprietary information on the PIE data for various burned fuel rods from Swiss reactors is available at PSI together with detailed information on the fuel operation and fuel design parameters. This allows for the validation of the fuel burnup calculations together with an assessment of the calculational vs. experimental uncertainty components of the obtained C/E results. Such studies were conducted for a KKG fuel rod sample (from a 15 × 15 fuel assembly irradiated during 3 cycles up to the sample final burnup above 50 GWd/tHM), and two types of uncertainties were assessed within this analysis:

- The uncertainties related to operating conditions, including boron concentration, moderator temperature, reactor power, etc. (the power and the moderator density were assumed fully correlated with the fuel and moderator temperatures in the underlying work described in Reference [8])
- The radiation (BU-)induced changes in the geometry (i.e., fuel pin position shift, moderator pin position shift, fuel pellet diameter increase, etc.)

As can be seen, these uncertainties represent the terms $\sigma_{OC}^{}$ and $\sigma_{BU-eff}^{}$ of Equation (2). The final assessments accepted for the given study are shown below in Table 6 (only the first figures are deemed significant). It shall be noticed that at present stage, the uncertainty components of Equation (2) are assumed to be uncorrelated. In reality, there might be some correlations between them, although they are not expected to be strong a priori, to the best knowledge of the authors. Stronger correlations would occur if one imposed into analysis certain reactor operational constraints, e.g., $k_{eff}=1$, at the reactor core follow simulations. However, in such cases the posterior uncertainties of the affected parameters are normally significantly reduced. Some representative illustrations on the example of the nuclear data evaluations can be found in References [35,36,37]. Furthermore, these types of uncertainties are most difficult for quantification and are already quite conservative by themselves, and therefore, questions on the uncertainties of these uncertainties or their correlations go far beyond the current work on the preliminary loading curves development.

#### 5.3.2. Technological Tolerances Impact

The impact of the PWR fuel technological and manufacturing parameter tolerances on the criticality calculations was analysed in Reference [38] with another PSI in-house tool “MTUQ” (Manufacturing and Technological Uncertainty Quantification). Taking into account only the fuel assembly-related uncertainties from the list of parameters used, the total $\sigma_{TP}^{}$ uncertainty component is assessed as only 10 pcm. In particular, the uncertainty components from all parameters considered in Figure 9 of Reference [38], except parameters 11 (Absorber Box—Inner boundary) and 13 (FA rack—Centre-to-centre distance), should be summed as random uncorrelated uncertainties, thus leading to a total uncertainty value limited by 10 pcm.

#### 5.3.3. Nuclear Data Uncertainty Impact

The uncertainties in the nuclear data employed in the calculations contribute to the uncertainty in the computed canister $k_{eff}$ values. Their impact was considered in the CASMO5 burnup calculations using SHARK-X methodology (see References [39,40] and references therein), providing the $\sigma_{ND-SNF}^{CASMO}\left(BU\right)$ estimation (uncertainties of the cross sections and the fission yields were taken into account), and in the MCNP6 criticality calculations using the Nuclear data Uncertainty Stochastic Sampling (NUSS) methodology to assess the $\sigma_{ND-Keff}^{MCNP}\left(BU\right)$ component (see References [39] and [29]; uncertainties of the thermal scattering (S(α,β)) data were ignored (see details and discussions in Reference [41]). The approach to estimate $\sigma_{ND-SNF}^{CASMO}\left(BU\right)$ was explained also in Reference [29]: “The nuclear data uncertainty propagation in CASMO depletion calculations, resulting in the spread of the SNF composition, was done using SHARK-X tool. The obtained set of the different SNF compositions was further translated to the SERPENT decay module for the decay simulation and finally provided to the MCNP6 models of the disposal canister to compute the spread of the $k_{eff}$ values due to the spread of the SNF compositions, using the nominal ENDF/B-VII.1 ND files.” Interested readers can find alternative ways of assessing the uncertainties associated with depletion calculations, for example, in References [42,43].

**Figure 7:**
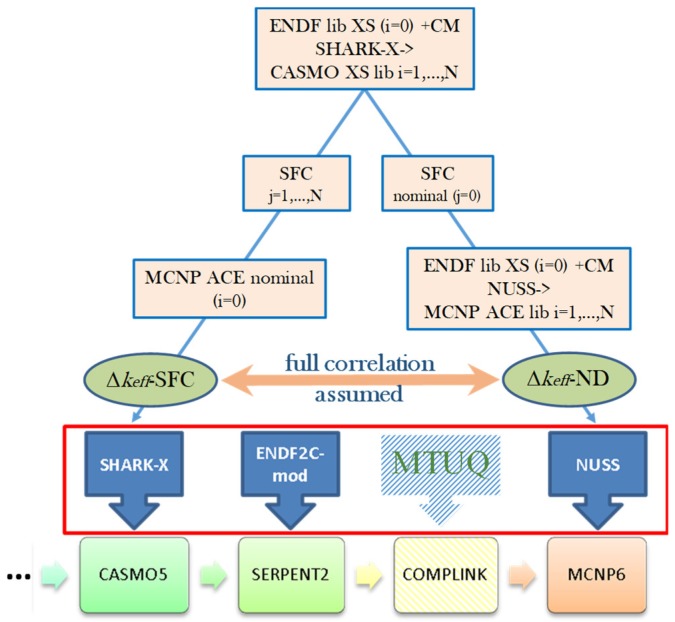
The presently employed nuclear data (ND) stochastic sampling methodology (“XS” is cross sections).

For an additional illustration, Figure 7 shows the scheme of the ND-related uncertainties (given as covariance matrices (CM)) propagation in compliance with the flowchart in Figure 3.

The Monte Carlo sampling method employed to obtain the estimated uncertainty in $k_{eff}$ requires a large number of calculations and has thus been realised only for the 4.94 w/o fuel enrichment case so far. 

**Figure 8:**
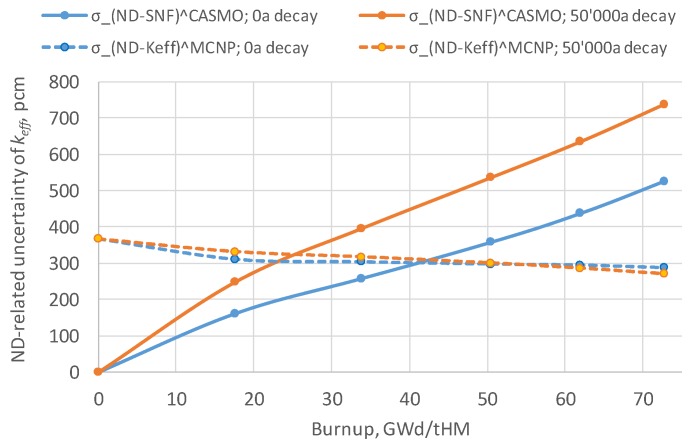
The estimated nuclear data-related uncertainties of $k_{eff}$.

Figure 8 shows the estimated $\sigma_{ND-SNF}^{CASMO}\left(BU\right)$ and $\sigma_{ND-Keff}^{MCNP}$ values for the fuel just after discharge and after 50,000 years of decay; uncertainties from all AC and FP are considered. The direct effect from the nuclear data in the MCNP6 calculation, $\sigma_{ND-Keff}^{MCNP}$, is similar in both periods and decreases slightly with the burnup level attained. The indirect effect of the nuclear data contained in the isotopic uncertainties, $\sigma_{ND-SNF}^{CASMO}\left(BU\right)$, increases with decay time and grows with burnup. These observations are valid for UO_2_ fuel and for the employed ENDF/B-VII.1 CM. Details of the calculations performed are given in Reference [39].

The decrease of the $\sigma_{ND-Keff}^{MCNP}$ uncertainty component with burnup is consistent with the results presented earlier in Reference [44], where even the net effect (uncertainty propagations through both the depletion and criticality calculations) of the ND uncertainties originating from the ENDF/B-VII.1 library was also found to decrease with burnup for UO_2_ fuel (see [44] and note that the impact of the fission yields uncertainties was not accounted for in [44], while in the given work the fission yields’ contribution is taken into account [39]). The decreasing uncertainty can potentially be explained by the decrease in the contribution of U-235 cross section uncertainties with burnup and probably with some spectrum-related effects (see also comments provided in Reference [29] for Figure 6 of Reference [29]).

It should be noted that the uncertainty components $\sigma_{ND-SNF}^{CASMO}$ and $\sigma_{ND-Keff}^{MCNP}$ must be correlated since the underlying nuclear data are the same for the independent estimations performed for both components. However, the correlation level has not been assessed. In the ideal case, all calculations should be done in a single set using the same original perturbation factors for the nuclear data in both the depletion and the criticality calculations; however, this will require significant additional computational burdens. Therefore, it will be conservative to assume a full correlation between both components and thus to estimate the total ND-related $k_{eff}$ component according to Equation (3). More advanced assessments are planned for the near future with the latest release of the ENDF/B library (ENDF/B-VIII), including the analysis of the ND-related correlations, as proposed in References [10,29].

Next, to be on the conservative side, the total ND-related uncertainty will be composed of the $\sigma_{ND-SNF}^{CASMO}$ component corresponding to 50,000 years of cooling and the $\sigma_{ND-Keff}^{MCNP}$ corresponding to zero cooling time (Figure 8).

#### 5.3.4. Long‑Term Nuclide Evolution

The performance of the decay module of the SERPENT2 code and the employed nuclear data library was investigated and benchmarked in Reference [18]. Later, the impact of the nuclear data uncertainty on the decay calculations realised with the SERPENT2 code was studied by perturbing the decay data with a modified version of the ENDF2C tool [39,45]. The main outcome shows an impact of $\sigma_{T_{1/2}}\;\approx$ 15 pcm on the $k_{eff}$ for the studied load.

#### 5.3.5. MCNP Monte Carlo Uncertainty

The Monte Carlo statistical uncertainty $\sigma_{MC}^{}$ of the calculations for the loading curve analysis was approximately ± 25 pcm, which is sufficiently low. 

### 5.4. Summary of Bounding Burnup Distributions and Random Uncertainties 

The analysed random uncertainty components are summarised in Table 7 (adapted from Reference [29]) together with the total sum of the uncertainties, $\sigma_{tot\;}^{}$, which will be used in Equation (1). It is important to note that the total uncertainty is burnup-dependent due to the burnup dependency of the components $\sigma_{ND}^{}$, $\sigma_{OP}^{}$ and $\sigma_{BU-eff}^{}$.

**Figure 9:**
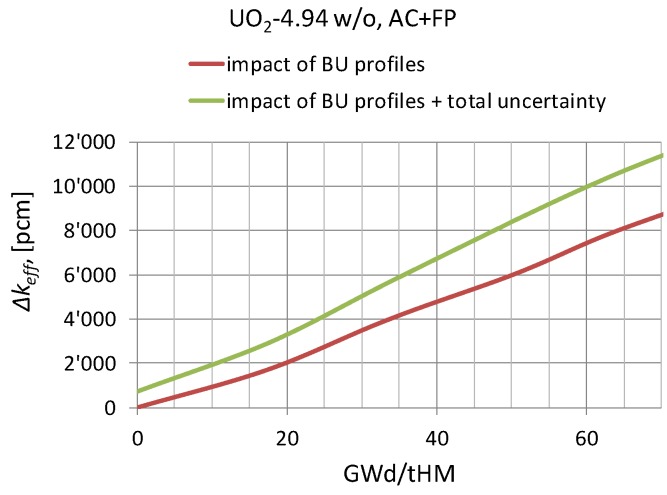
The impact of the burnup profiles and the total uncertainty on the canister $k_{eff}$ value.

To better illustrate the impact of the considered burnup profile penalties and the uncertainty components, Figure 9 shows the results obtained for the case of AC+FP based on the data reported in Table 6, Figure 8 and Table 7 (the red line is the direct sum of the axial (Table 2) and radial (Table 4) effects; adapted from Reference [29]).

## 6. Loading Curves with Combined Uncertainties Effects

The final target of this work was to assess a minimum average burnup for individual fuel assemblies required for a full loading of the disposal canister without exceeding the defined upper subcritical limit. This goal is accomplished by the development of specific loading curves for discharged spent fuels, where the initial enrichment and final burnup of a fuel bundle will function as the acceptance criteria for the loading of the disposal canister. 

The development of the curve is done as follows: the left part of Equation (1) is represented by a curve depending on the burnup, while the right part of Equation (1) is a constant corresponding to the given USL value. If the burnup-dependent curve of Equation (1) and the burnup-independent USL line intersect, the burnup at the point of the intersection becomes the point on the loading curve corresponding to the given fuel enrichment. If the burnup-dependent curve of Equation (1) is always below the USL value, then for the given enrichment, the minimum burnup equals zero on the loading curve. As presented in Section 4.1, for the PSI CSE methodology using the MCNP code in conjunction with ENDF/B-VII.1 and assuming the “administrative margin” equals 5000 pcm, the USL value is defined according to Equation (1) as 0.99339 − 0.05000 = 0.94339.

**Figure 10:**
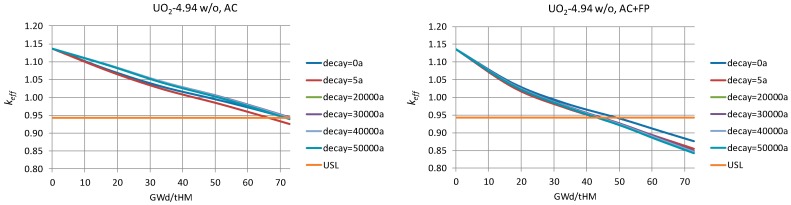
An illustration of the determination of the minimum burnup required for fuel to meet the Upper Subcritical Limit (USL) criticality safety criteria for AC (**left**) and AC+FP (**right**).

To give an outlook on the general behaviour of the curve over the burnup, Figure 10 shows the examples for the case of AC (left) and AC+FP (right) corresponding to the highest of all the considered enrichments, 4.94 w/o. 

It can be seen again that the most conservative case for the credit of AC+FP is zero cooling time after discharge, while in the case of only AC, the most conservative results generally correspond to the cooling time of about 30,000 years (in particular, for the AC cases with 2.50 w/o and 3.50 w/o enrichments, the highest $k_{eff}$ results took place at zero cooling time, and in the case of 3.50 w/o enrichment, at 20,000 years cooling time; in all other cases, the most conservative cooling time was 30,000 years).

The computed minimum required burnups for each particular enrichment are shown in Table 8.

**Figure 11:**
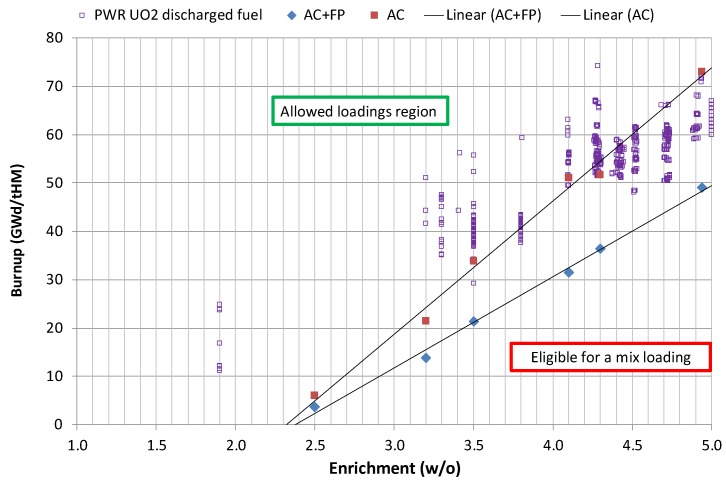
The preliminary loading curves with all the conservative effects for discharged spent fuel (valid only for the criticality safety criteria, and subject to discussed important assumptions).

The loading curves are then derived by applying the data from Table 8 as shown in Figure 11 (adapted from Reference [29]).

Some examples on the KKG discharged fuel assemblies’ characteristics (viz., initial enrichment and discharged burnup) are also demonstrated in Figure 11. From these results, it becomes clear that the given canister design meets the criticality safety criteria if the burnup credit is based on the AC+FP approach. A potential exception could be, for instance, the 5 w/o enriched FAs if they were irradiated for a lower number of cycles than designed, e.g., discharged once-burned from a last operated core. In reality, the last cycles can be loaded with a lower enriched fuel to avoid the inefficient use of fissile material. 

It can be seen from Figure 11 that, for the given USL value and for the case of UO_2_, 4.94 w/o fuel, the saving in terms of the minimum required burnup as compared between the cases of AC+FP vs. AC is about 24 GWd/tHM (this has been illustrated in more detail in Reference [29]). This result was obtained by comparing the cases which take into account the bounding burnup profiles and also all uncertainties listed in Table 7. Basically, the same effect (23 GWd/tHM) can be observed for the case when only bounding burnup profiles are taken into account with no uncertainties contribution. A much weaker effect would be obtained (16 GWd/tHM) if the calculations were done with nominal burnup profiles instead of the bounding ones. Finally, a comparison between two values of the administrative margin was also shown in Reference [29]: for the conventional value of 5000 pcm and for a reduced one of 2000 pcm. This illustration allowed the prediction of what saving in the minimum burnup requirement could be achieved, provided the administrative margin could hypothetically be relaxed to 2000 pcm. The extra saving for such a case was estimated as approximately 12 GWd/tHM. 

## 7. Outlook and Discussion 

The classical practice for criticality safety calculations for applications out of the reactor has relied on the fresh fuel assumption. The advantages of taking credit for changes in the actinide compositions after irradiation and the build-up of fission products in the spent fuel, with a net effect of decreasing the $k_{eff}$ of the systems formed by burned fuel assemblies, have been given major attention during the last decades. Initially applied to spent fuel pools and fuel dissolution facilities and later to transport and dry storage, burnup credit has also been used in waste disposal applications in the USA, Sweden and Finland. The waste management applications in these three countries have been reviewed by the regulators, are under review or are under development respectively.

In line with this, a preliminary criticality assessment study has been performed taking into account the burnup credit within the PSI/Nagra BUCSS-R research project recently conducted at PSI. Ways for further methodological improvements and the verification of the present results are discussed below.

### 7.1. Ways to Improve the Reference BUCSS-R Methodology 

Based on the analyses performed so far, it can be concluded that the most significant uncertainty components at the current stage are

- the nuclear data (ND) combined impact ($\sigma_{ND}^{}$),
- the operating conditions ($\sigma_{OC}^{}$), and
- the radiation-/burnup-induced geometry changes ($\sigma_{BU-eff}^{}$).

Obviously, it can be recommended at first to verify, in particular, the most significant contributors to the loading curve burnup penalties in future work (although future studies should not be limited to only the listed components since other parameters can appear significant in more comprehensive simulations). Also, it would be relevant to assess the SIMULATE-3/BOHR/CASMO5 methodology component approximations, namely the infinite lattice calculations of the pin-wise SNF compositions with CASMO5 code. With respect to this limitation, the transition to the SIMULATE5 code in combination with another Studsvik code “SNF” (in quotation marks to distinguish from the earlier spent nuclear fuel abbreviation) could provide intra-assembly pin-wise isotopic distributions from the real core-follow calculations, as discussed in the next section. Such work is currently ongoing at PSI outside the present research collaboration with NAGRA.

Furthermore, a comparison of the current methodologies, which can be classified as a combination of the best estimate plus uncertainties (BEPU) approach with certain conservative assumptions, against more traditional methodologies can be recommended. In particular, the comparison of the BEPU SNF compositions versus analogue data but with application of the so-called isotopic correction factors [13] obtained from the validation studies with the post-irradiation examination (PIE) data should be considered.

Finally, the inclusion of the correlation analysis in the CSE methodology, as discussed in Reference [29], should be considered, since it would lead to more accurate and probably less penalising loading curves. 

### 7.2. Alternative Advanced Way to Derive the Loading Curves under Development and Verification

The presently developed BUCSS-R calculation scheme is considered at PSI to be sufficiently detailed and accurate for the construction of the BEPU-type loading curves, provided that statistically representative samples can be obtained for FAs with all considered fuel types and enrichments. A drawback of this system is that it is rather demanding in terms of calculation resources. Important to mention is that the SIMULATE/BOHR/CASMO calculations required for the BUCSS-R scheme are basically not needed for any other type of simulation, so they are practically solely dedicated to the BUCSS-R methodology for the loading curve derivations (As outlined before, the BOHR methodology is also useful for the PIE data analysis, but in that case, only important FA segments can be calculated, which drastically reduces the computational demands.). Since the CASMO calculations generally have to be redone every time an updated/new version of the CASMO code or/and its cross section library are released, such a method for the derivation of the loading curves becomes costly and inefficient.

**Figure 12:**
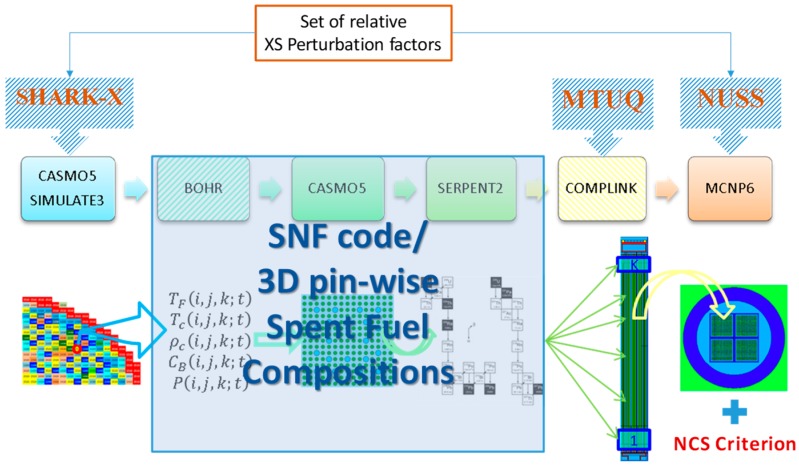
An alternative computational scheme based on the “SNF” code integration into the CMSYS system.

However, an alternative way to derive the loading curves recently became available based on the exploitation of the advanced features of the additional CMS code “SNF”, which can be seamlessly integrated into the PSI CMSYS system. A description of the new methodology developed at PSI outside the BUCSS-R project, which is based on the CASMO/SIMULATE/SNF/COMPLINK/MCNP system of codes and which was named the CS_2_M method has been presented in Reference [46], together with examples of its trial application. Thus, the concept of the new and more advanced approach for the loading curve derivation is to rely on the nominal and validated CMSYS CASMO/SIMULATE core-follow models and calculations, with no need for complementary CASMO calculations to derive the SFC. All required information can now be obtained directly from the CASMO/SIMULATE results with the help of the “SNF” code, as illustrated schematically in Figure 12 (see Reference [47] for details on the relative perturbation factors).

Furthermore, as already mentioned, it is recommended in the future to use the SIMULATE5 code, as it has a more accurate inter-assembly SFC characterisation capability. In doing so, it will be in principle possible to extract the SFC for every individual fuel assembly ever operated in Switzerland and thus to avoid all undesirable approximations to realise the BEPU calculations. The uncertainty propagation can be done with the same tools as used before in the BUCSS-R scheme. This is illustrated with the shaded boxes in the top part of Figure 12.

Therefore, although the new CS2M approach is also time-consuming, it does not require any complementary and unnecessary simplified assessments, and for that reason, it is more efficient and transparent for verification and validation. 

**Figure 13:**
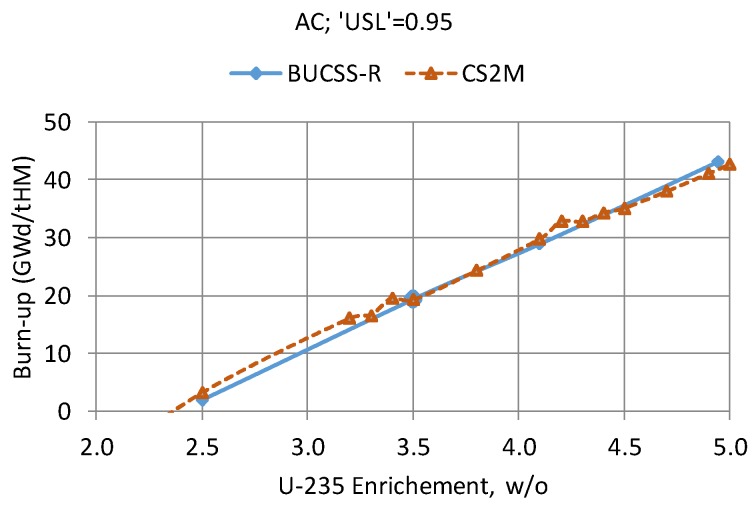
A comparison of the test loading curves obtained with the reference BUCSS-R and the advanced CS2M schemes (for the same simplified conditions).

Finally, since both approaches being developed at PSI to derive the loading curves for the Swiss reactor spent fuel assemblies differ from the more conventional “conservative” approaches (see Section 4 “Group Discussions” of Reference [48]), it is definitely an advantageous situation at PSI that two different methods can be compared, and in such way, the resulting loading curves can be verified with high confidence. In line with this, the results of the work in Reference [46] and of the BUCSS-R assessments presented in this paper have been compared, as demonstrated in Figure 13. For a consistent comparison, the BUCSS-R results were reevaluated for this particular graph, using the same conditions as were applied in Reference [46]: no uncertainty components and no bounding burnup profiles are taken into account in this test exercise, i.e., only the reference/nominal burnup profiles are considered. As can be seen, the results are in good agreement. Future work will be focused on the extended and more representative verification studies using both BUCSS-R and CS2M calculation schemes.

## 8. Conclusions

This work was oriented towards deriving the preliminary loading curves for the SNF disposal canisters to be used in a Swiss deep geological repository currently being planned by Nagra. This paper contains a description of the applied methodology and presents the main findings and results.

In the initial stage of the study, bounding fuel type analyses using representative fuel assemblies operated in the Swiss PWR plant KKG were performed. The UO_2_, MOX and ERU fuels were analysed using the highest enrichments used to date and operated to the highest burnups to properly assess their behaviour. As detailed as possible, intra-assembly SNF compositions have been used in the MCNP criticality calculations based on the results of calculations corresponding to realistic cycle operating conditions extracted from the validated PSI core management models. The case of PWR UO_2_ has been confirmed to be the most limiting, and consequently, the loading curve analysis was done for this fuel.

The methodology applied for the loading curve derivation integrated the outcome of the standard PSI criticality safety validation procedure with the estimated penalties on the computed due to the uncertainties in the nuclear data, fuel assembly design parameters and operating conditions as well as radiation-induced changes in the fuel assembly geometry. Furthermore, bounding axial and radial burnup profiles and the most reactive fuel loading configuration in the canister, in terms of penalising radial tilt, were taken into account accordingly.

The loading curves obtained for the reference disposal canister (as illustrated in Figure 11) show what minimum average fuel assembly burnup is required for the given original fuel enrichment of fresh fuel assemblies so that the $k_{eff}$ of the canister would comply with the imposed criticality safety criterion.

The loading curves presented were obtained for a reference disposal canister design provided by Nagra in the course of the project. However, Nagra is exploring various options for the selection of the materials and design concepts for the canister, which may require a reevaluation of the loading curves. A preliminary study demonstrating the plausibility of the alternative canister designs can be found in Reference [2]. 

The loading curves presented in this work show that the AC credit approach would not be sufficient to meet the USL criticality safety criterion for a non-mixed loading with fuel with an initial enrichment above approximately 3.5 w/o, while the AC+FP approach justifies the applicability of the canister design considered for the safe disposal of spent nuclear fuel with all the existing enrichments with required minimum burnups.

A postulated hypothetical case consisting of FAs with 5 w/o initial enrichment and relatively low burnup would be the only exception to fitting the loading criteria; however, this case belongs only to a theoretical last core discharge, where, in reality, a lower enriched fuel should be employed. The option of some canisters being not fully loaded could be an alternative approach but would be challenging from another point of view (logistics and cost optimisation). Theoretically, mixed configurations of SNF with different burnup levels could also be a solution for the most problematic cases.

The loading curves must be treated as preliminary since there is still room for improvement in the assessment of different components of Equations (1)–(3). However, valuable findings have already been obtained concerning the identification of the most problematic scenarios and loading schemes, which will serve as guiding information for the next phases of the research and development at PSI and Nagra, in particular for the optimisation of the canister design and the mixed burnup/fuel assembly design loading schemes.

Among the topics for further improvement of the BUCSS-R methodology (with respect to only the criticality safety and burnup credit assessments), other aspects can be proposed for consideration, such as the extension of the analysed FA sample to yield 95%/95% tolerance bounds for the loading curves (i.e., to safely cover the potential uncertainties from the operating condition variations); a refinement of the treatment of the uncertainties with respect to the burnup axial and radial profiles, allowing a consistent “Total Monte Carlo” assessment (“seamless” calculations of both depletion/decay and criticality models using the same ND perturbation factors, as outlined in Figure 12); and the assessment of long-term scenarios with canister and fuel evolution and degradation. In addition, an improvement of the nuclear criticality safety criterion based on a detailed analysis of the nuclear data-related uncertainties and correlations, which can be done, e.g., with NUSS [10,49], has been proposed in References [10,29] and should be further explored.

In parallel, assessments of an alternative option for the currently used BUCSS-R methodology are ongoing independently at PSI, for which Studsvik’s code “SNF” is integrated into the CS_2_M scheme [46] to substitute the BOHR component of the BUCSS-R scheme. At present, the new approach is basically used for the verification of the base BOHR/BUCSS-R results; however, if confirmed to be more efficient, this new calculation scheme can replace the original one in the future studies at PSI.

## Figures and Tables

**Table 1:** The $k_{eff}$ values for the canister configurations with fresh fuel.

Assumed Conditions	4 UO_2_	4 ERU	4 MOX	1 MOX + 3 UO_2_	1 Empty + 3 UO_2_
Helium filled	0.21146	0.20772	0.26259	0.20743	0.17861
Flooded/centred	1.09513	1.08022	0.96180	1.07035	1.02971
Flooded/inwards	1.12903	1.11227	0.98601	1.10079	1.04864
Flooded/outwards	1.04355	1.02920	0.91990	1.02301	0.99541

**Table 2:** The $\Delta k_{eff}$ penalty due to the bounding axial burnup profiles for the case of 4.94 w/o, pcm.

	Discharge Burnup (GWd/tHM)									
Time (a)	17.61 *		33.82		50.47		61.92		72.75	
	AC	AC+FP	AC	AC+FP	AC	AC+FP	AC	AC+FP	AC	AC+FP
0	-	-	983	1792	2359	3390	3604	4880	4737	6223
5	-	-	1273	2203	2752	4042	4141	5642	5322	7144
20,000	-	-	1209	2445	2886	4946	4571	7113	6210	9078
30,000	-	-	1197	2445	2930	4968	4675	7218	6414	9236
40,000	-	-	1225	2544	2950	5064	4729	7307	6538	9493
50,000	-	-	1213	2533	2993	5154	4901	7381	6693	9609

* At the first discharge burnup of 17.61 GWd/tHM, the nominal axial burnup profile is actually more reactive than the penalising one.

**Table 3:** The $\Delta k_{eff}$ penalty due to the bounding axial burnup profiles for the case of 3.5 w/o, pcm.

	Discharge Burnup (GWd/tHM)							
Time (a)	18.9		33.66		45.25		56.15	
	AC	AC+FP	AC	AC+FP	AC	AC+FP	AC	AC+FP
0	401	1037	1703	2306	3711	4705	4910	6211
5	592	1387	1931	2860	4129	5575	5484	7252
20,000	587	1769	2143	3603	4904	7184	6808	9472
30,000	577	1905	2248	3698	4974	7258	6982	9647
40,000	674	1919	2274	3760	5064	7411	7271	9944
50,000	634	2026	2350	3826	5294	7593	7401	10,144

**Table 4:** The $\Delta k_{eff}$ penalty due to the bounding radial burnup profiles for the case of 4.94 w/o, pcm.

	Discharge Burnup (GWd/tHM)									
Time (a)	17.61		33.82		50.47		61.92		72.75	
	AC	AC+FP	AC	AC+FP	AC	AC+FP	AC	AC+FP	AC	AC+FP
0	1380	1729	2008	2230	2100	2656	2449	2829	2375	2820
5	1464	1947	2187	2475	2364	3016	2584	3268	2548	3103
20,000	1246	1832	2194	2743	2553	3550	2917	3959	2926	3858
30,000	1202	1831	2185	2821	2601	3652	3009	3952	3040	3883
40,000	1208	1860	2225	2815	2630	3663	3028	3976	3121	3973
50,000	1260	1860	2219	2888	2670	3751	3073	3997	3102	4045

**Table 5:** The $\Delta k_{eff}$ penalty due to the bounding radial burnup profiles for the case of 3.5 w/o, pcm.

	Discharge Burnup (GWd/tHM)							
Time (a)	18.9		33.66		45.25		56.15	
	AC	AC+FP	AC	AC+FP	AC	AC+FP	AC	AC+FP
0	1966	2339	2278	2511	2866	3210	2780	3163
5	2140	2575	2248	2975	2928	3710	2972	3617
20,000	2150	2761	2633	3553	3567	4429	3642	4601
30,000	2107	2799	2603	3657	3577	4572	3701	4617
40,000	2151	2770	2666	3677	3586	4624	3851	4750
50,000	2198	2911	2754	3733	3754	4638	3879	4826

**Table 6:** The available uncertainty data on the operating conditions and the BU-induced geometry changes, pcm.

Burnup (GWd/tHM)	0.0–17.6	17.6–33.8	33.8–50.5
Operating conditions	100	400	500
BU-induced changes	200	200	700

**Table 7:** A summary of all the random uncertainty components, pcm.

Burnup (GWd/tHM)	$\sigma_{ND}^{}$	$\sigma_{OP}^{}$	$\sigma_{BU-eff}^{}$	$\sigma_{TP}^{}$	$\sigma_{T_{1/2}}\;$	$\sigma_{MC}^{}$	$1\sigma_{tot\;}^{}$	$2\sigma_{tot\;}^{}$
0	367	0	0	10	15	25	368	737
17.61	560	100	200	10	15	25	604	1208
33.82	700	400	200	10	15	25	831	1662
50.47	834	500	700	10	15	25	1199	2397
61.92	930	500	700	10	15	25	1267	2534
72.75	1026	500	700	10	15	25	1339	2679

**Table 8:** The minimum burnup required for meeting the criticality safety criteria.

Enrichment w/o	AC	AC+FP
1.90	0	0
2.50	~6.0 *	3.7
3.20	21.5	13.8
3.50	33.9	21.4
4.10	~51.0 *	31.5
4.30	~51.7 *	~36.4 *
4.94	~73.0 *	49.1

* These values are based on extrapolations or involve certain (minor) interpolations.
